# Ribulose-1,5-Bisphosphate Carboxylase/Oxygenase (RubisCO) Is Essential for Growth of the Methanotroph Methylococcus capsulatus Strain Bath

**DOI:** 10.1128/AEM.00881-21

**Published:** 2021-08-26

**Authors:** Calvin A. Henard, Chao Wu, Wei Xiong, Jessica M. Henard, Brett Davidheiser-Kroll, Fabini D. Orata, Michael T. Guarnieri

**Affiliations:** a Department of Biological Sciences and BioDiscovery Institute, University of North Texas, Denton, Texas, USA; b Biosciences Center, National Renewable Energy Laboratory, Golden, Colorado, USA; c Geological Sciences Department, University of Colorado, Boulder, Colorado, USA; d Department of Chemical and Materials Engineering, University of Alberta, Edmonton, Alberta, Canada; e Department of Biological Sciences, University of Alberta, Edmonton, Alberta, Canada; University of Illinois at Urbana-Champaign

**Keywords:** methanotroph, autotroph, RubisCO, greenhouse gas, methane, biogas, one-carbon metabolism

## Abstract

The ribulose-1,5-bisphosphate carboxylase/oxygenase (RubisCO) enzyme found in plants, algae, and an array of autotrophic bacteria is also encoded by a subset of methanotrophs, but its role in these microbes has largely remained elusive. In this study, we showed that CO_2_ was requisite for RubisCO-encoding Methylococcus capsulatus strain Bath growth in a bioreactor with continuous influent and effluent gas flow. RNA sequencing identified active transcription of several carboxylating enzymes, including key enzymes of the Calvin and serine cycles, that could mediate CO_2_ assimilation during cultivation with both CH_4_ and CO_2_ as carbon sources. Marker exchange mutagenesis of M. capsulatus Bath genes encoding key enzymes of potential CO_2_-assimilating metabolic pathways indicated that a complete serine cycle is not required, whereas RubisCO is essential for growth of this bacterium. ^13^CO_2_ tracer analysis showed that CH_4_ and CO_2_ enter overlapping anaplerotic pathways and implicated RubisCO as the primary enzyme mediating CO_2_ assimilation in *M. capsulatus* Bath. Notably, we quantified the relative abundance of 3-phosphoglycerate and ribulose-1,5-bisphosphate ^13^C isotopes, which supported that RubisCO-produced 3-phosphoglycerate is primarily converted to ribulose-1-5-bisphosphate via the oxidative pentose phosphate pathway in *M. capsulatus* Bath. Collectively, our data establish that RubisCO and CO_2_ play essential roles in *M. capsulatus* Bath metabolism. This study expands the known capacity of methanotrophs to fix CO_2_ via RubisCO, which may play a more pivotal role in the Earth’s biogeochemical carbon cycling and greenhouse gas regulation than previously recognized. Further, *M. capsulatus* Bath and other CO_2_-assimilating methanotrophs represent excellent candidates for use in the bioconversion of biogas waste streams that consist of both CH_4_ and CO_2_.

**IMPORTANCE** The importance of RubisCO and CO_2_ in *M. capsulatus* Bath metabolism is unclear. In this study, we demonstrated that both CO_2_ and RubisCO are essential for *M. capsulatus* Bath growth. ^13^CO_2_ tracing experiments supported that RubisCO mediates CO_2_ fixation and that a noncanonical Calvin cycle is active in this organism. Our study provides insights into the expanding knowledge of methanotroph metabolism and implicates dually CH_4_/CO_2_-utilizing bacteria as more important players in the biogeochemical carbon cycle than previously appreciated. In addition, *M. capsulatus* and other methanotrophs with CO_2_ assimilation capacity represent candidate organisms for the development of biotechnologies to mitigate the two most abundant greenhouse gases, CH_4_ and CO_2_.

## INTRODUCTION

Methanotrophs are metabolically unique bacteria that are capable of utilizing CH_4_ as a carbon and/or energy source (1). These microbes occupy an array of ecological niches across the globe and are vital in regulating atmospheric CH_4_ by either preventing its release into the atmosphere or directly sequestering it from the air (2). Three metabolic modes of CH_4_ assimilation have been described in phylogenetically diverse methanotrophic bacteria (3–5). These include (i) the ribulose monophosphate (RuMP) cycle, primarily utilized by gammaproteobacterial methanotrophs; (ii) the serine cycle, primarily utilized by alphaproteobacterial methanotrophs; and (iii) the Calvin-Basham-Benson (CBB) cycle, utilized by the verrucomicrobial and candidate phylum NC10 methanotrophs. The oxidation of CH_4_ is essential for energy generation in all methanotrophs, but various CH_4_ oxidation products, including formaldehyde, formate, and/or CO_2_, serve as carbon sources in these bacteria, depending on their single-carbon assimilation pathway.

Methylococcus capsulatus Bath is a gammaproteobacterial methanotroph that has served as a model methanotrophic bacterium; much of what has been learned about biological methane conversion, including the metabolic pathways and enzymes involved in CH_4_ oxidation and assimilation, are based on seminal research utilizing this bacterium (6–10). Several independent laboratories have observed that *M. capsulatus* Bath exhibits inconsistent growth if exogenous CO_2_ is not included in the gas phase (11, 12). This phenotype can be circumvented in continuous culture if the gas flow rate is significantly decreased (13, 14). Many hypotheses related to this phenotype have been proposed, including that supplying exogenous CO_2_ supports the serine cycle in this organism under certain conditions, which is based on observations that CO_2_ supplementation increases growth of alphaproteobacterial methanotrophs (15). Another hypothesis is that, in contrast to the majority of culturable alphaproteobacterial and gammaproteobacterial methanotrophs, *M. capsulatus* Bath requires exogenous CO_2_ for growth. This hypothesis is supported by the presence of RubisCO and phosphoribulokinase in this organism (16, 17), which would enable a complete CBB cycle commonly used by chemoautotrophic bacteria and phototrophs for CO_2_ assimilation. Notably, CO_2_ assimilation by *M. capsulatus* Bath has been observed in the presence of an energy source such as CH_4_ or H_2_ (11, 18). Furthermore, *M. capsulatus* Bath autotrophic growth with H_2_ as an energy source has been demonstrated on solid medium in a sealed growth vessel, but attempts to cultivate it autotrophically in liquid culture have failed (19). Thus, the roles of RubisCO and the CBB cycle in *M. capsulatus* Bath central metabolism and physiology remain elusive. In this study, we revisited the capacity of *M. capsulatus* Bath to utilize CO_2_ and the potential role of RubisCO in CO_2_ fixation and the central metabolism of this organism. Using reverse genetics approaches, we show that RubisCO-mediated CO_2_ assimilation is essential for the growth of *M. capsulatus* Bath. Furthermore, ^13^CO_2_ isotopic tracing analyses indicated that a CBB cycle variant is active in this bacterium and highlight extensive overlap between CH_4_ and CO_2_ utilization pathways. These results establish that RubisCO and CO_2_ are central to *M. capsulatus* Bath metabolism and provide insight into the CO_2_-dependent methanotrophy occurring in this bacterium.

## RESULTS

### CO_2_ assimilation improves growth of *M. capsulatus* Bath.

**FIG 1:**
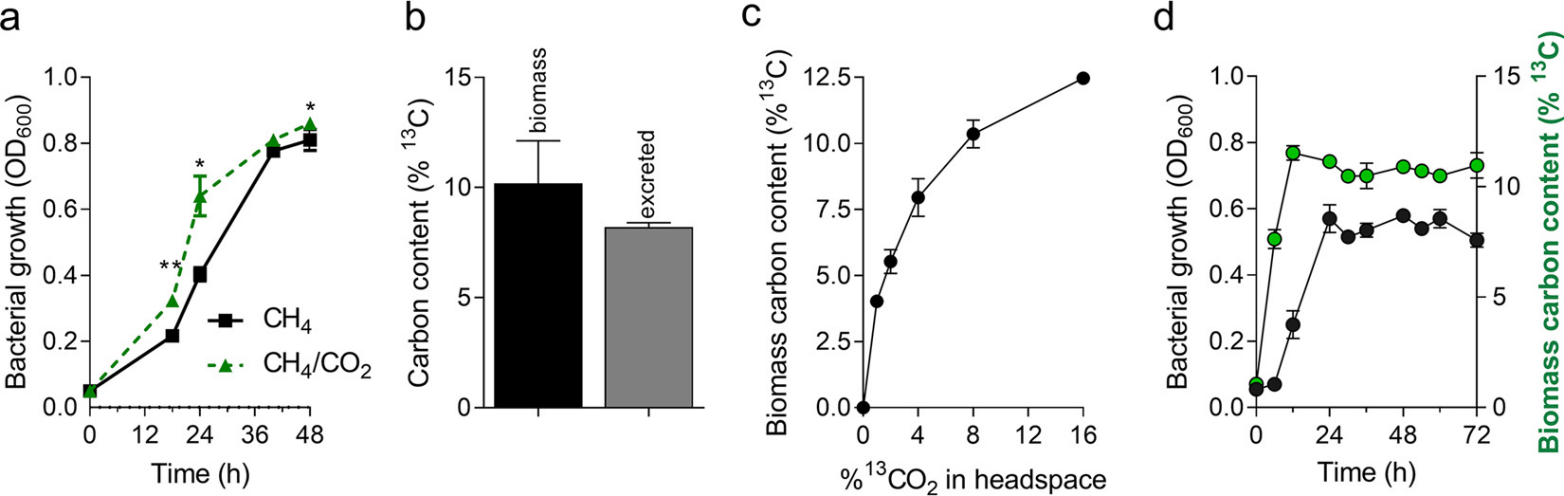
CO_2_ supplementation enhances *M. capsulatus* Bath growth in sealed vials. (a) *M. capsulatus* Bath growth based on optical density at 600 nm (OD_600_) in sealed serum vials supplemented with 20% CH_4_ and 8% CO_2_ in air (green dotted line) or 20% CH_4_ in air only (black solid line). (b) Percentage of biomass or excreted compounds derived from ^13^CO_2_ determined by isotopic element analysis after 48 h of cultivation. (c) Percentage of biomass derived from ^13^CO_2_ determined by isotopic element analysis after 48 h of cultivation with increasing concentrations (1 to 16%) of ^13^CO_2_. (d) Culture density (OD_600_) and ^13^C-derived biomass during *M. capsulatus* Bath cultivation with 20% CH_4_ and 8% ^13^CO_2_ in the gas phase. Data represent the mean ± standard deviation of 4 to 6 biological replicates from two independent experiments. **, *P* ≤ 0.01; *, P ≤ 0.05, determined by unpaired Student’s *t* test.

Initially, we tested the effect of CO_2_ addition on *M. capsulatus* Bath growth with CH_4_ as the primary carbon source. CO_2_ supplementation to the gas phase of sealed serum vials slightly improved *M. capsulatus* Bath growth compared to that with CH_4_ alone (Fig. 1a). Isotopic elemental analysis of *M. capsulatus* Bath cultured in serum vials with 8% ^13^CO_2_ in the gas phase mixture containing 20% CH_4_ in air showed that a significant percentage (>10%) of *M. capsulatus* Bath biomass and excreted compounds in the culture medium were derived from exogenous CO_2_ (Fig. 1b), which was 4 to 10× higher than CO_2_ assimilated via basal carboxylation reactions in Escherichia coli or in the related gammaproteobacterial methanotroph Methylotuvimicrobium alcaliphilum 20Z^R^, which does not encode RubisCO (see Fig. S1a in the supplemental material). Titrating ^13^CO_2_ in the gas phase indicated that maximum CO_2_ assimilation by *M. capsulatus* Bath was limited below 8% CO_2_ (vol/vol) (Fig. 1c), which is stoichiometrically consistent with the total biomass generated and the maximum percentage derived from CO_2_ under these growth conditions. ^13^CO_2_ was assimilated immediately upon introduction to the culture (Fig. 1d), and a positive correlation between growth and ^13^CO_2_ incorporation into biomass was observed during active growth (Fig. 1d); thus, *M. capsulatus* Bath displays concurrent CH_4_ and CO_2_ metabolisms. We note the possibility that the percentage of total biomass derived from CO_2_ is potentially even higher than that determined by elemental analysis, as unlabeled CH_4_-derived CO_2_ evolved into the headspace is likely assimilated by the bacteria under these growth conditions.

### CO_2_ is required for *M. capsulatus* Bath growth in an unsealed bioreactor with continuous gas supply.

**FIG 2:**
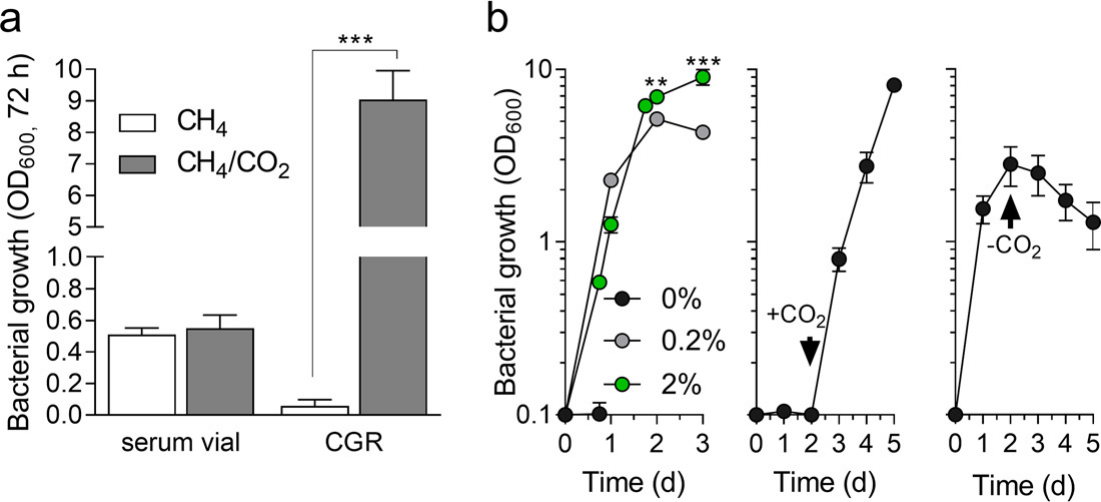
*M. capsulatus* Bath CO_2_-dependent growth. (a) Growth of *M. capsulatus* Bath in a sealed serum vial or a continuous gas reactor (CGR) with 20% CH_4_ in air only (white bar) or supplemented with 2% CO_2_ (gray bar). (b) *M. capsulatus* Bath growth in a CGR supplied with 20% CH_4_ and 0.2% or 2% CO_2_ in air at 1 volume gas mixture/volume medium/min (left). After 2 days of cultivation with 20% CH_4_ in air, 2% CO_2_ was added (+CO_2_, middle) or removed (−CO_2_, right) from the gas phase. Data represent the mean ± standard deviation of 4 to 6 biological replicates from two independent experiments. ***, *P* ≤ 0.001; ** P ≤ 0.01, determined by unpaired Student’s *t* test.

M. capsulatus Bath can be cultivated in sealed serum vials when CH_4_ is the only carbon source supplied in the vial gas phase (Fig. 2a). However, we observed that *M. capsulatus* Bath did not grow in an unsealed bioreactor with continuous CH_4_ supply (20% CH_4_ in air; 1 volume gas/volume vessel/min) unless CO_2_ was also provided in the gas mixture (Fig. 2a and b), a phenotype not observed for M. alcaliphilum 20Z^R^ (Fig. S1b). This CO_2_-requiring growth phenotype was confirmed with the additional *M. capsulatus* strains *M. capsulatus* Bath (ATCC 33009) and M. capsulatus Texas (ATCC 19069) to rule out potential strain variation artifacts known to exist within methylotrophic bacterial laboratory strains (Fig. S1c) (20). Similar growth kinetics were measured in bioreactors during logarithmic growth between cultures supplied with 0.2% CO_2_ and 2% CO_2_ in 20% CH_4_ and air; however, 0.2% CO_2_ became limiting as the culture density increased, restricting the maximum culture density to ∼50% of that observed with 2% CO_2_ supplementation (Fig. 2b). We successfully cultivated *M. capsulatus* Bath without CO_2_ if the gas flow rate was reduced (20% CH_4_ in air; 0.1 volume gas/volume vessel/min), but the bacteria showed significantly slower growth kinetics and reduced culture density (optical density at 600 nm [OD_600_] of ∼4 after 7 days of cultivation; see Fig. S1d) compared to faster gas flow rates when CO_2_ was requisite (Fig. 2b). Collectively, these data suggest that CH_4_-derived CO_2_ is evolved and can support *M. capsulatus* Bath growth under some cultivation conditions (sealed serum vials and unsealed vessels with low CH_4_ supply rates) but is stripped from an unsealed bioreactor with high flow rates. *M. capsulatus* Bath is widely used for the production of single-cell protein and is cultivated at industrial scale in proprietary U-loop bioreactors supplied with natural gas and oxygen/air mixtures (21–23). Notably, the U-loop bioreactor is sealed, such that CO_2_ evolved from the gas fermentation is only vented to maintain gas partial pressures that ensure optimal gas solubility. Thus, *M. capsulatus* Bath likely uses CH_4_-derived CO_2_ during U-loop reactor cultivation.

### RubisCO is an essential *M. capsulatus* Bath gene.

**FIG 3:**
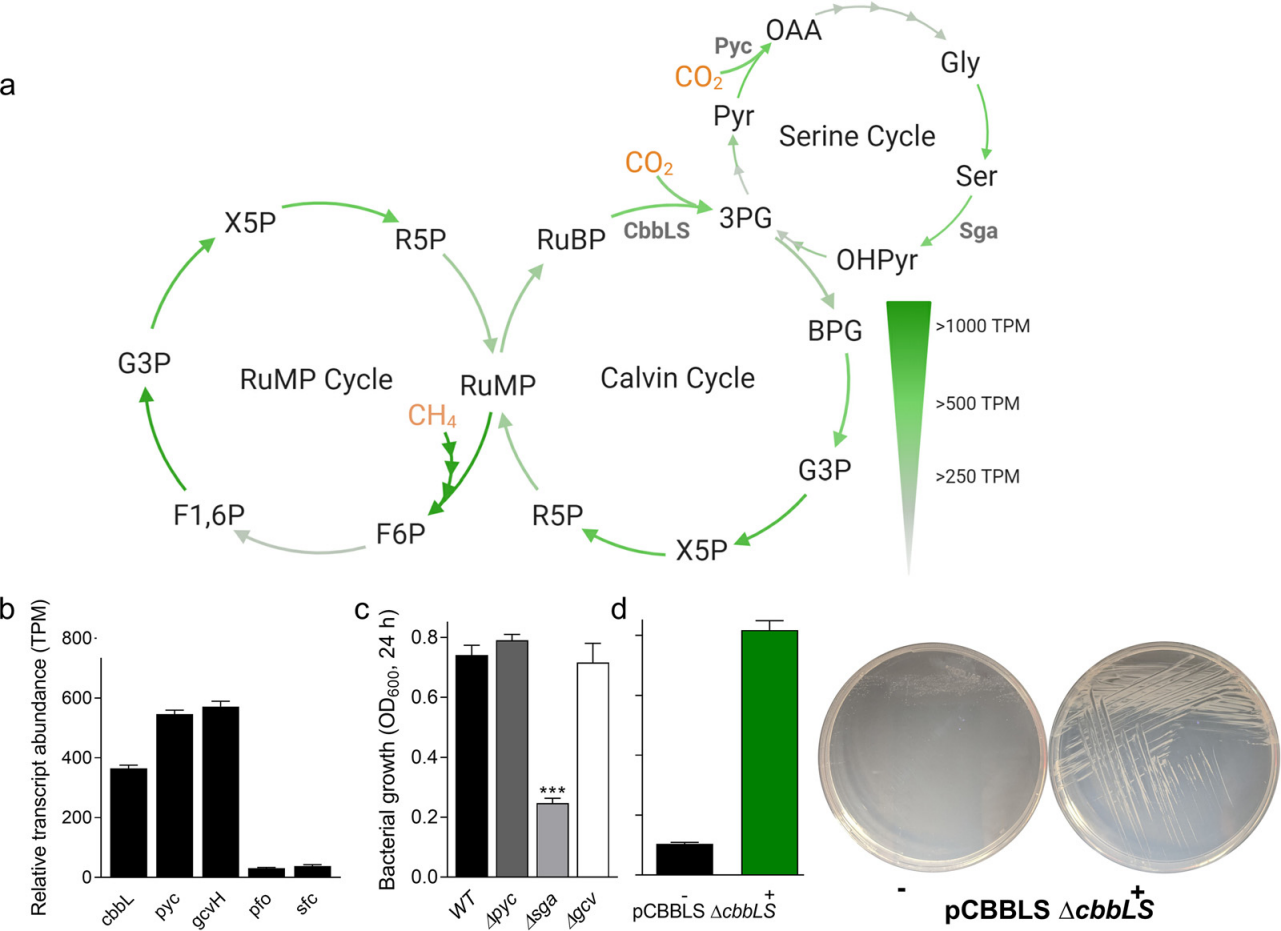
RubisCO-dependent growth of *M. capsulatus* Bath. (a) Relative abundance of *M. capsulatus* Bath transcripts for enzymes of the ribulose monophosphate pathway (RuMP), Calvin-Basham-Benson (CBB) cycle, and serine cycle C_1_ assimilation routes during cultivation in a bioreactor with continuous influent and effluent gas. (b) Relative transcript abundance of *M. capsulatus* Bath RubisCO large subunit (*cbbL*), pyruvate carboxylase (*pyc*), glycine cleavage protein H (*gcvH*), pyruvate::ferredoxin oxidoreductase (*pfo*), and malic enzyme (*sfc*) putative carboxylating enzymes. (c) Cultivation of pyruvate carboxylase (Δ*pyc*, dark gray bar), serine-glyoxylate amino transferase (Δ*sga*, light gray bar), and glycine cleavage (Δ*gcv*, white bar) knockout strains compared to wild-type (black bar) *M. capsulatus* Bath in sealed serum vials. (d) Cultivation of the conditional RubisCO knockout strain (pCbbLS Δ*cbbLS*::Gm^r^) in a serum vial (left) or on solid NMS medium (right) with (+) or without (−) RubisCO induction. Data represent the mean ± standard deviation of 4 biological replicates from two independent experiments (b to d) or a representative experiment (a and d). ***, *P* ≤ 0.001 compared to WT, determined by unpaired Student’s *t* test.

In *M. capsulatus* Bath genes encoding RubisCO, phosphoribulokinase, overlapping RuMP and CBB cycle enzymes, and serine cycle enzymes are transcribed at variable levels during active growth with continuous dual CH_4_ and CO_2_ supply (Fig. 3a; see also Table S1 in the supplemental material). Relative transcript abundances of carboxylating enzymes that could mediate CO_2_ assimilation by *M. capsulatus* Bath, including the serine cycle’s carboxylating enzyme, pyruvate carboxylase (*pyc*), and the H-protein component of the putative carboxylating glycine cleavage enzyme, were 1.5-fold higher than the RubisCO large subunit transcript (Fig. 3b). Two other potential carboxylating enzymes, the pyruvate::ferredoxin oxidoreductase (*pfo*) and malic enzyme (*sfc*), exhibited significantly lower relative transcription compared to the other carboxylating enzyme transcripts (<50 transcripts per million [TPM]).

Pyruvate carboxylase (Δ*pyc*), serine-glyoxylate aminotransferase (Δ*sga*), or glycine cleavage (Δ*gcv*) knockout strains obtained using marker exchange mutagenesis exhibited similar or retarded (Δ*sga*) growth in serum vials compared to that of wild-type *M. capsulatus* Bath (Fig. 3c). The comparable growth of the Δ*pyc* and Δ*gcv* knockout strains to that of the wild type indicated that these enzymes are not essential for CO_2_-dependent growth of this organism. Interestingly, we observed a growth defect by the Δ*sga* knockout strain, a similar phenotype to that recently observed in *M. alcaliphilum* 20Z^R^, which does not require exogenously supplied CO_2_ for growth (24). Collectively, the Δ*sga* and Δ*pyc* knockout strain phenotypes suggest that the serine-glyoxylate aminotransferase is involved in *M. capsulatus* Bath oxaloacetate and/or glyoxylate conversion, but that a canonical serine cycle that would be dependent on a phosphoenolpyruvate or pyruvate carboxylase is not essential for *M. capsulatus* Bath CO_2_-dependent growth. We note the possibility that *M. capsulatus* Bath could encode an alternative enzyme to facilitate a complete serine cycle, although such an enzyme is not readily identified in the available genome.

We next hypothesized that CO_2_ assimilation by this organism is mediated by RubisCO, which has been shown to be active during growth on CH_4_ (11). To test this hypothesis, we attempted but were unable to obtain a RubisCO knockout strain (Δ*cbbLS*) using similar genetic approaches to those used in generating the Δ*pyc*, Δ*gcv*, and Δ*sga* knockout strains, suggesting that RubisCO-encoding genes are essential. However, leveraging custom genetic tools for inducible gene expression (25), we developed a conditional RubisCO knockout strain with inducible, ectopic homologous RubisCO expression (pCBBLS Δ*cbbLS*). The pCBBLS Δ*cbbLS* strain exhibited similar growth to that of wild-type *M. capsulatus* Bath in both liquid and solid medium supplemented with the RubisCO inducer anhydrotetracycline (aTc). However, we observed no bacterial growth in liquid medium and significantly reduced growth on solid medium in the absence of RubisCO induction (Fig. 3d), supporting the conclusion that RubisCO is required for *M. capsulatus* Bath growth. Leaky *cbbLS* transcription could have enabled the limited growth we observed on solid medium, since we have previously measured leaky expression from the P*_tet_* promoter in the absence of aTc induction in *M. capsulatus* Bath (26).

### CH_4_ and CO_2_ enter overlapping *M. capsulatus* Bath central metabolic pathways.

**FIG 4:**
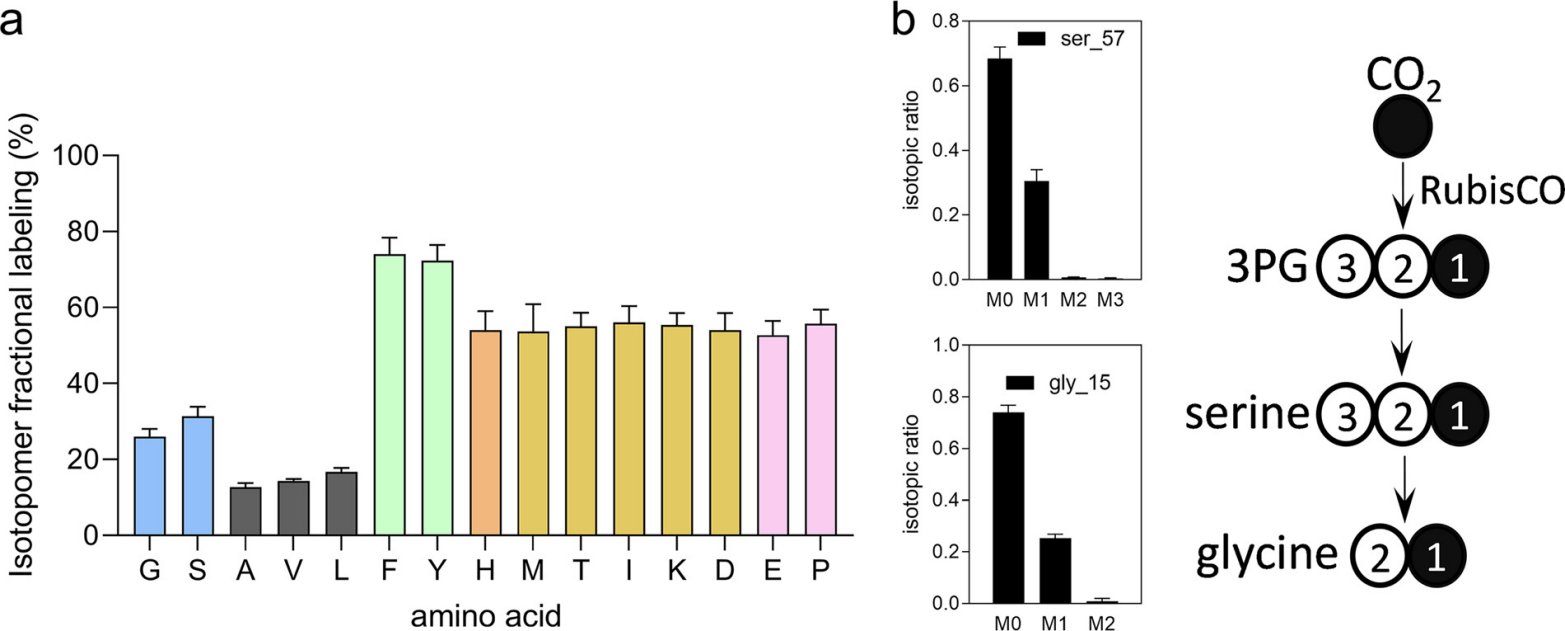
^13^CO_2_ tracing and amino acid isotopomers implicate *M. capsulatus* Bath RubisCO in CO_2_ fixation. Isotopomer analysis of proteinogenic amino acids in cultures grown with 20% unlabeled CH_4_ and 8% ^13^CO_2_. (a) Total % ^13^C labeling and (b) isotopic labeling of phosphoglycerate-derived amino acids. Amino acids are color coded based on their respective metabolite precursors, as follows: 3-phosphoglycerate (light blue), ribose-5-phosphate (orange), phosphoenolpyruvate and erythrose-4-phosphate (green), pyruvate (gray), oxaloacetate (yellow), and α-ketoglutarate (pink). Data represent the mean mass isotopomer distribution vector of three biological replicates.

We next cultivated *M. capsulatus* Bath with ^12^CH_4_ and ^13^CO_2_ to identify enzymes mediating CO_2_ fixation and metabolites derived from CO_2_. ^13^C-labeling patterns of derivatized amino acids indicated that central metabolites derived from CO_2_ enter core intermediary metabolic pathways, including the Embden-Meyerhof-Parnas (EMP) glycolytic pathway, the pentose phosphate pathway, and the tricarboxylic acid (TCA) cycle in this organism (Fig. 4a; see also Fig. S2 and Table S2 in the supplemental material). 3-Phosphoglycerate (3PG)-derived glycine and serine incorporated ^13^C exclusively in the C-1 position, which implicated RubisCO as the primary carboxylating enzyme responsible for CO_2_-derived 3PG production (Fig. 4b). In agreement with the genetic analyses, these amino acid isotopomer labeling patterns confirmed that RubisCO is the primary enzyme mediating CO_2_ assimilation in *M. capsulatus* Bath and excluded carboxylation by pyruvate carboxylase, glycine synthase, malate synthase, pyruvate::ferredoxin oxidoreductase, or acetyl-coenzyme A (CoA) carboxylase as primary CO_2_ assimilation reactions in this bacterium.

**FIG 5:**
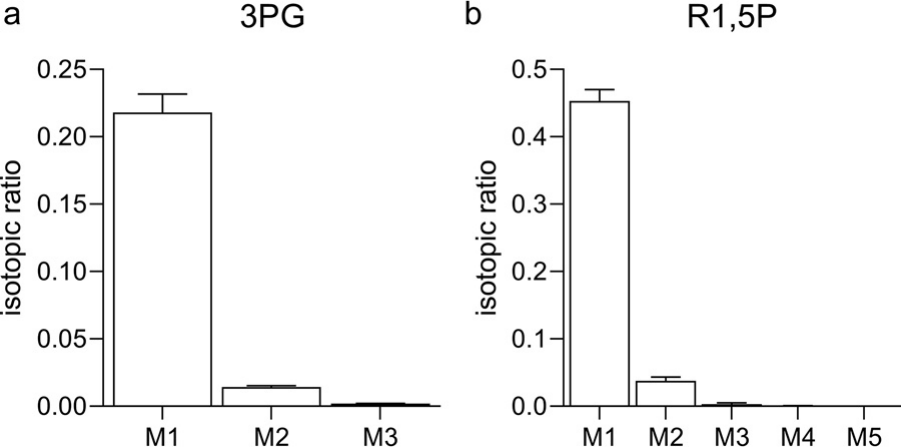
A dual one-carbon ribulose monophosphate/bisphosphate cycle is active in *M. capsulatus* Bath. Isotopomer analysis of the RubisCO carboxylation product (a) 3-phosphoglycerate (3PG) and substrate (b) ribulose-1,5-bisphosphate (R1,5P) in cultures supplied with 8% ^13^CO_2_ for 4 h. Data represent the mean mass isotopomer distribution vector ± standard deviation of three biological replicates.

**FIG 6:**
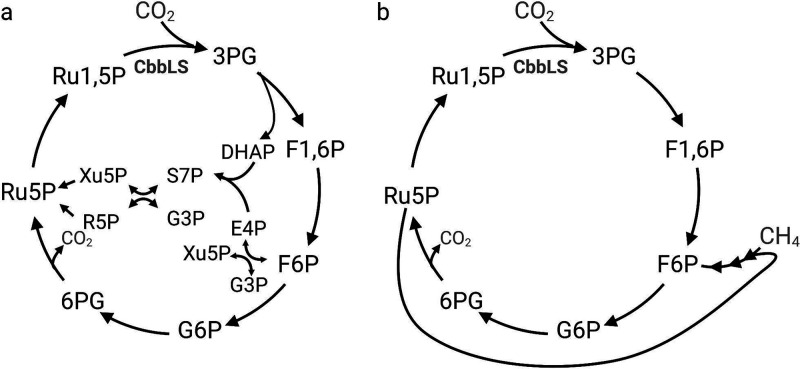
Proposed dual one-carbon ribulose monophosphate/bisphosphate cycle in *M. capsulatus* Bath. (a) Potential biosynthetic routes for ribulose 1,5-bisphosphate generation in *M. capsulatus* Bath. (b) *M. capsulatus* Bath CBB cycle variant deduced from ^13^CO_2_ tracing and metabolite analyses. 3PG, 3-phosphoglycerate; G3P, glyceraldehyde 3-phosphate; DHAP, dihydroxyacetone phosphate; F1,6P, fructose 1,6-bisphosphate; F6P, fructose 6-phosphate; E4P, erythrose 4-phosphate; S7P, sedoheptulose 7-phosphate; R5P, ribose 5-phosphate; Xu5P, xylulose 5-phosphate; Ru5P, ribulose 5-phosphate; R1,5P, ribulose 1,5-bisphosphate; G6P, glucose-6-phosphate; 6PG, 6-phosphogluconate.

To confirm that 3PG is produced via RubisCO, as the serine and glycine isotopomer labeling patterns suggested, we quantified 3PG isotopomers directly. Consistent with RubisCO-dependent carboxylation of ribulose-1,5-bisphosphate (R1,5P), we measured the M1 3PG isotopomer after 4 h of labeling and found it to be 22% of the total 3PG pool (Fig. 5a). R1,5P isotopomers were also measured to determine if RubisCO-derived 3PG is used for regeneration of the RubisCO substrate (labeled R1,5P) or if RubisCO-derived 3PG enters the EMP pathway and is completely converted to pyruvate in this bacterium (unlabeled R1,5P). The M1 R1,5P isotopomer was enriched (∼45%), confirming that R1,5P is derived from both CH_4_ and CO_2_ and that a CBB cycle is active in *M. capsulatus* Bath (Fig. 5b). Surprisingly, we detected few of the doubly labeled M2 R1,5P isotopes (<5% of total isotopomers) that would be expected if ribulose-5-phosphate (Ru5P) is derived via carbon rearrangements occurring in the nonoxidative branch of the pentose phosphate pathway. The enriched M1 R1,5P isotopomer with limited detection of the M2 isotopomer indicates that R1,5P is regenerated via the oxidative branch rather than via the nonoxidative branch of the pentose phosphate pathway, representing a noncanonical CBB cycle (Fig. 6). Collectively, isotopomer fingerprints of *M. capsulatus* central metabolites indicate that core intermediates are derived from both CH_4_ and CO_2_ carbon sources, supporting a high degree of metabolic plasticity and an essential interplay between CH_4_ and CO_2_ metabolism that engenders a novel, dual C_1_-fixing RuMP/RuBP pathway in this organism.

## DISCUSSION

It has been 40 years since the initial discovery that *M. capsulatus* Bath possesses RubisCO activity and derives a portion of its biomass from CO_2_ (11, 18); however, the importance of CO_2_ assimilation and the RubisCO enzyme in *M. capsulatus* Bath metabolism has remain unclear. In this study, we have provided evidence that both CO_2_ and RubisCO are required for optimal *M. capsulatus* Bath metabolism and growth. Our results indicate that RubisCO assimilates CO_2_ to produce 3PG and that a CBB cycle is active in this bacterium.

Our results expand upon the known carbon assimilation routes utilized by diverse methanotrophic bacteria. It is generally accepted that the majority of gammaproteobacterial methanotrophs utilize CH_4_ as their sole carbon and energy source, whereas alphaproteobacterial methanotrophs assimilate CH_4_ derivatives and CO_2_ via the serine cycle (1, 3). Recent evidence suggests that verrucomicrobial and candidate phylum NC10 methanotrophs utilize CH_4_ as an energy source and CO_2_ as a sole carbon source (27–31). Additionally, the verrucomicrobial Methylacidiphilum spp. have been demonstrated to grow autotrophically when using hydrogen as an energy source (30). Despite this mixotrophic metabolism, neither we nor others have successfully cultivated *M. capsulatus* Bath autotrophically in liquid medium. Our results show that *M. capsulatus* Bath is a chemoorganoautotroph that strongly prefers CH_4_ as an energy source and requires both CH_4_ and CO_2_ as carbon sources. Further research is needed to understand why some methanotrophs encode RubisCO and assimilate CO_2_ via a CBB cycle while others do not, but the metabolic plasticity afforded by the presence of hydrogenases, methane monooxygenases, and RubisCO may allow these microbes to inhabit and contribute to primary productivity in diverse habitats (2, 32, 33).

This study demonstrates that CO_2_ is essential for *M. capsulatus* Bath growth. *M. capsulatus* Bath actively expresses several putative carboxylases that could enable CO_2_ assimilation, but our data conclusively show that RubisCO is essential for *M. capsulatus* Bath under the experimental conditions utilized here. Other carboxylases that are part of the serine cycle and the recently discovered autotrophic reductive glycine pathway (34) that could mediate CO_2_ fixation are also encoded by this organism. However, genetic analyses and ^13^CO_2_ isotopic tracing excluded these pathways as primary routes of CO_2_ assimilation in this bacterium. In contrast, ∼70% of the biomass of Methylosinus trichosporium OB3b, an alphaproteobacterial methanotroph that uses the serine cycle for CH_4_-derived carbon assimilation, is derived from CO_2_ (15). These results underscore that the serine cycle can be a primary route of CO_2_ assimilation in both methylotrophs and methanotrophs that use this pathway for methanol or CH_4_ assimilation, respectively. These alternative CO_2_ fixation pathways may play important metabolic roles for *M. capsulatus* in specific environments with variable CO_2_ availability. Notably, carboxylases and the associated biochemical pathways present in *M. capsulatus* Bath represent rational metabolic engineering targets for increasing the CO_2_ utilization capacity of this methanotroph for complete conversion of biogas or nonphotosynthetic CO_2_ capture.

Several CBB cycle variants and RubisCO-dependent pathways function in nature (35, 36). The isotopomer labeling patterns of 3PG and R1,5P (both singly ^13^C-labeled) suggest that a CBB cycle variant that overlaps with the known primary CH_4_ flux through the oxidative pentose phosphate pathway (oxPPP)/Entner-Doudoroff pathway is functional in M. capsulatus Bath (10). In the canonical transketolase-dependent CBB cycle, ribose-5-phosphate (R5P) and xylulose-5-phosphate (Xu5P) are produced from glyceraldehyde-3-phosphate and sedoheptulose-7-phosphate by transketolase during carbon rearrangement reactions of the nonoxidative pentose phosphate pathway (noPPP; Fig. 6a). M2 and M3 doubly and triply labeled R1,5P isotopomers would be expected if Ru5P were derived from the noPPP R5P and Xu5P metabolites due to carbon rearrangements, similarly to what we observed in the isotopomer distributions of the aromatic amino acids phenylalanine and tyrosine, which are derived from the noPPP metabolite erythrose-4-phosphate (Fig. 4a). However, R1,5P isotopomers primarily consisted of singly labeled M1 isotopes (Fig. 5b), which supports that the phosphoribulokinase substrate Ru5P is primarily derived from 6-phosphogluconate (6PG) formed in the oxPPP (Fig. 6b). This in an intriguing observation, given that decarboxylation of 6PG to Ru5P would represent a futile CBB cycle. It is possible that flux through oxPPP to Ru5P supports cellular reducing power, since two NADPH reducing equivalents are generated in this pathway. The reductive power generated by oxPPP is associated with boosting antioxidant defenses in many bacteria and eukaryotes (37–40); thus, increased flux through this pathway could be essential for *M. capsulatus* Bath to maintain redox homeostasis and/or to support the increased NADPH required for CO_2_ assimilation. Furthermore, the increased growth kinetics and overall biomass yield we observed (Fig. 1) support that RubisCO and CO_2_ also serve a biosynthetic role, likely providing an alternative route of anaplerotic metabolites that enables metabolic plasticity for *M. capsulatus* Bath. Additional experimentation is required to validate the putative CBB cycle variant, determine its potential contribution to cellular energetics, and its relationship with CH_4_ assimilation, since Ru5P also serves as the substrate for formaldehyde condensation in this methanotroph.

*M. capsulatus* Bath is an obligate aerobe, so its RubisCO presumably exhibits both carboxylase and oxygenase activities during growth in the presence of CO_2_ and O_2_, generating phosphoglycerate and phosphoglycolate from these substrates, respectively. Indeed, phosphoglycolate phosphatase activity in *M. capsulatus* Bath cell-free extracts and bacterial phosphoglycolate utilization were previously demonstrated (18). We observed significant transcription of a putative phosphoglycolate phosphatase gene (MCA_RS12655) that likely encodes the enzyme mediating conversion and utilization of RubisCO-generated phosphoglycolate during active growth with dual CH_4_/CO_2_ supply (see Table S1 in the supplemental material). *M. capsulatus* Bath also encodes a putative glycolate oxidase (MCA_RS07375 to MCA_RS07380) that catalyzes the conversion of glycolate to glyoxylate. Glyoxylate entry into the serine cycle would complete a unique metabolic pathway in this methanotroph, a serine cycle variant of the phosphoglycolate salvage pathway recently described in the chemolithotroph Cupriavidus necator (41). In support of this hypothesis, the growth defect observed by the Δ*sga* mutant strain that cannot convert glyoxylate to glycine could be due to a deficiency in the capacity to metabolize phosphoglycolate. Thus, the production of phosphoglycolate and its downstream metabolism may contribute, in part, to the essentiality of RubisCO that is independent of the observed *M. capsulatus* Bath requirement for CO_2_.

The essentiality of *M. capsulatus* Bath RubisCO could also be partially attributed to a role in controlling intracellular R1,5P levels. Accumulation of R1,5P significantly inhibits the bacterial growth of E. coli expressing heterologous phosphoribulokinase (42). Furthermore, RubisCO has been shown to play important roles in the regulation of both redox and R1,5P levels in purple nonsulfur bacteria (43, 44). Notably, recent evidence indicates that one-carbon assimilation in the methylotroph Methylorubrum extorquens is regulated by an R1,5P-responsive allosteric transcriptional activator, QscR, that induces expression of serine cycle enzymes (45). *M. capsulatus* Bath encodes a QscR homolog (MCA_RS14905) with 41% amino acid identity to the M. extorquens protein. If R1,5P plays a similar regulatory role or causes toxicity in *M. capsulatus* Bath, the accumulation of R1,5P in the absence of RubisCO could preclude isolation of a RubisCO-null mutant. Thus, additional inquiry into the role of R1,5P in *M. capsulatus* Bath physiology is warranted.

The *M. capsulatus* Bath dual CH_4_/CO_2_ metabolism described here provides additional insight into one-carbon metabolism and the potential evolutionary relationship between methanotrophy and RubisCO-mediated autotrophy. The extensive overlap in metabolites produced and converting enzymes required for sole fixation of each carbon source in related methanotrophic and autotrophic bacteria supports this evolutionary relationship. Notably, this dual CH_4_/CO_2_ metabolism designates *M. capsulatus* Bath as a promising biocatalyst for simultaneous mitigation and valorization of the two most abundant and harmful atmospheric greenhouse gases (46, 47).

## MATERIALS AND METHODS

### Methanotroph cultivation.

Bacterial strains used in this study are shown in Table 1. The primary Methylococcus capsulatus Bath strain used during the course of these investigations was obtained from the Mary Lidstrom laboratory at the University of Washington. Additional *M. capsulatus* strains were obtained from the American Type Culture Collection (Bath [ATCC 33009] and Texas [ATCC 19069]). *M. capsulatus* Bath cultures were routinely maintained with nitrate mineral salts (NMS) solid medium in stainless steel gas chambers supplied with 20% CH_4_ in the gas phase, as previously described (26). Methylotuvimicrobium alcaliphilum 20Z^R^ was cultured in modified NMS medium containing 3% NaCl and carbonate buffer as described previously (48). Strains were grown in 150-ml vials containing 30 ml of growth medium. After inoculation with plate-derived biomass, vials were crimped with gray butyl stoppers to create gas-tight seals. CH_4_ was added to the headspace to reach a final CH_4_ of 20% in air (vol/vol), and cultures were incubated at 37°C (*M. capsulatus*) or 30°C (*M. alcaliphilum*) at 200 rpm orbital shaking. Continuous gas cultivation was performed using a custom midthroughput gas fermentation reactor (MGFR) in 150-ml Kimax cultivation tubes fitted with stainless steel sparge stones. Culture aliquots (100 ml) were inoculated with plate-derived biomass and supplied with 20% CH_4_ in air (vol/vol) or 20% CH_4_/0.2 to 2% CO_2_ in air (vol/vol) at a flow rate of 1 volume gas/volume culture/min (vvm) premixed with gas-specific mass flow controllers. For the CH_4_ only cultivation of *M. alcaliphilum*, carbonate buffer was removed from the medium formulation, after which the pH of the medium was stabilized at 9.5 with KOH.

**TABLE 1:** Strains and plasmids

Strain or plasmid	Genotype or description^*a*^	Source or reference
Strains		
Methylococcus capsulatus strain Bath	Wild type	ATCC 33009
Methylococcus capsulatus strain Texas	Wild type	ATCC 19069
Methylococcus capsulatus strain Bath (ML)	Wild-type laboratory strain	Mary Lidstrom Laboratory
pCbbLS Δ*cbbLS*::Gm^r^	ML carrying the pCbbLS plasmid with MCA_RS13440 to MCA_RS13445 genes replaced with an FRT-flanked Gm^r^ cassette	This study
Δ*sga*::Gm^r^	MCA_RS06920 gene replaced with an FRT-flanked Gm^r^ cassette	This study
Δ*pyc*::Gm^r^	MCA_RS12165 to MCA_RS12170 genes replaced with an FRT-flanked Gm^r^ cassette	This study
Δ*gcv*::Gm^r^	MCA_RS01715 to MCA_RS01725 genes replaced with an FRT-flanked Gm^r^ cassette	This study
Escherichia coli strain Zymo 10B	F^−^ *mcrA* Δ(*mrr-hsdRMS-mcrBC*) Φ80*lacZ*ΔM15 Δ*lacX*74 *recA1 endA1 araD139* Δ(*ara leu*) 7697 *galU galK rpsL nupG*	Zymo Research
E. coli S17-1	Tp^r^ Sm^r^ *recA thi pro hsd*(r^−^m^+^)RP4-2-Tc::Mu::Km Tn*7*	ATCC 47055
Methylotuvimicrobium alcaliphilum 20Z^R^	Rifampin-resistant variant	(48)
Plasmids		
pCAH01	P*_tetA_ bla tetR* CoE1*ori* F1 *oriV oriT trfA ahp*	25
pCM184	Marker exchange mutagenesis vector	Addgene no. 46012 (50)
pPS856	Source of FRT-flanked *aacC* Gm^r^ cassette	49
pCMM433kanT	Source of *oriT* backbone for *pyc* and *sga* marker exchange mutagenesis vectors	51
pCbbLS	pCAH01 with *M. capsulatus cbbLS* (MCA2743–MCA2744)	This study

a Gm^r^, gentamicin resistance; Tp^r^, trimethoprim resistance; Sm^r^, streptomycin resistance; FRT, FLP recombination target.

### Knockout strain construction.

Primers used in this study are shown in Table 2. The *M. capsulatus* Bath *cbbLS* (MCA_RS13440 to MCA_RS13445) genes encoding the RubisCO large and small subunits, respectively, were amplified using oCAH644 and oCAH655 primers and cloned into pCAH01 via Gibson assembly to generate pRUB with inducible RubisCO expression under the control of the tetracycline promoter/operator. pRUB was transferred to *M. capsulatus* Bath via biparental conjugation using E. coli S17 as previously described (26). Genomic fragments (1,000 bp) flanking the *cbbLS*, pyruvate carboxylase (*pyc* [MCA_RS12165 to MCA_RS12170]), glycine cleavage aminomethyltransferase and glycine dehydrogenase components (*gcv*, [MCA_RS01715 to MCA_RS01725]), or serine-glyoxylate aminotransferase (*sga* [MCA_RS06920]) genes, an FLP recombination target (FRT)-flanked gentamicin resistance cassette from pPS856 (49), and a pCM184 (50) or pCM433kanT (51) fragment containing an origin of transfer (*oriT*) were amplified by PCR and assembled independently via Gibson assembly. The resulting marker exchange mutagenesis plasmids for generating *pyc*, *gcv*, or *sga* knockout strains were introduced into wild-type *M. capsulatus* Bath via biparental conjugation, and transformants were selected on solid NMS medium containing 30 μg/ml gentamicin. The *cbbLS* marker exchange mutagenesis plasmid was introduced to the pCbbLS-containing strain by biparental conjugation in the presence of the anhydrotetracycline (aTc) inducer. After mating, gentamicin-resistant clones were selected and maintained on NMS medium supplemented with aTc (0.5 μg/ml), gentamicin (30 μg/ml), and kanamycin (50 μg/ml). Positive transformants were PCR genotyped for the absence of wild-type *pyc*, *gcv*, or *sga* loci using the primers oCAH1233/oCAH1234, oCAH151/oCAH152, or oCAH1215/oCAH1216, respectively. The absence of the *cbbLS* genes and the presence of pCbbLS were confirmed via PCR using the primers oCAH964/oCAH965 and oCAH172/oCAH173, respectively, followed by sequence verification. Conditional growth of the pCbbLS Δ*cbbL*::Gm^r^ strain was performed on NMS solid medium with or without 0.5 μg/ml aTc (RubisCO induction) supplementation.

**TABLE 2:** Primers

Primer purpose or name	Sequence^*b*^
*M. capsulatus* Bath *cbbLS* knockout construct and confirmation
oCAH062 pCM184 R	CTAGACGTCAGGTGGCAC
oCAH069 pCM184 F	TCACTAGAGGATCCAGCC
oCAH884 *cbbL* 1kbUP F	ccgaaaagtgccacctgacgtctagCATGCGTTTGGTCCAGGC
oCAH885 *cbbL* 1kbUP R	tgaagctaattcgGGTTTTCTCCTACTCGTTTTGC
oCAH886 FRT-Gm^R^-FRT F	gtaggagaaaaccCGAATTAGCTTCAAAAGC
oCAH887 FRT-Gm^R^-FRT R	ggttaccgcagagCGAATTGGGGATCTTGAAG
oCAH888 *cbbS* 1kbDWN F	gatccccaattcgCTCTGCGGTAACCCCGGG
oCAH889 *cbbS* 1kbDWN R	cctggtcggctggatcctctagtgaTGTCGTGGAAAGCGGAGC
oCAH964 *cbb* KO F	CAGCGCTTATAGTGCCTCGC
oCAH965 *cbb* KO R	CTGGATGTTGTCCATGAAGAAGCC
*M. capsulatus* Bath *pyc* knockout construct and confirmation
oCAH1124 pCM433 R	TTTCCTGCATTTGCCTGTTTC
oCAH1125 pCM433 F	GAATGAATCACCGATACG
oCAH1132 *pyc* 1kbUP F	aaacaggcaaatgcaggaaaAATTTGCCCAGTGCCTCGGC
oCAH1026 *pyc* 1kbUP R	aagatccccaattcgGCGGGAAGTTTCAGGTTAGCGACAA
oCAH1027 FRT-Gm^R^-FRT F	cctgaaacttcccgcCGAATTGGGGATCTTGAAG
oCAH1028 FRT-Gm^R^-FRT R	cgcaattcgcgaggaCGAATTAGCTTCAAAAGC
oCAH1029 *pyc* 1kbDWN F	tttgaagctaattcgTCCTCGCGAATTGCGCTCTTTC
oCAH1133 *pyc* 1kbDWN R	cgcgtatcggtgattcattcAGCAGCTGGTGGACGGTC
oCAH1233 *pyc* KO F	TTTGTCGCTAACCTGAAACTTCCCG
oCAH1234 *pyc* KO R	TCCCGAAAGAGCGCAATTCG
*M. capsulatus* Bath *sga* knockout construct and confirmation
oCAH1193 *sga* 1kbUP F	aaacaggcaaatgcaggaaaATGCTTTTCCATCCCGAAC
oCAH1194 *sga* 1kbUP R	gatccccaattcgCAAATTCACCTTGTGTTATAGG
oCAH1195 FRT-Gm^R^-FRT F	caaggtgaatttgCGAATTGGGGATCTTGAAG
oCAH1196 FRT-Gm^R^-FRT R	gaggtccggcggaCGAATTAGCTTCAAAAGC
oCAH1197 *sga* 1kbDWN F	tgaagctaattcgTCCGCCGGACCTCGCCGG
oCAH1198 *sga* 1kbDWN R	cgcgtatcggtgattcattcGCCCATCGCGATCCTCGTCTCC
oCAH1215 *sga* KO F	GTTCCCATCCGTGCCTCTAC
oCAH1216 Gm R	GCCTTCGACCAAGAAGCGGT
oCAH1237 *sga* KO R	CAACCATCAGTCTCCGGCGA
*M. capsulatus* Bath *gcv* knockout construct and confirmation^*a*^
oCAH145 gcv 1kbUP F	actggaaacaggcaaatgcaggaaaGTCGATACCGAACTCGCC
oCAH146 gcv 1kbUP R	gatccccaattcgGGTGTCGTGTCCTCGAAAC
oCAH147 gcv FRT-Gm^R^-FRT F	ggacacgacaccCGAATTGGGGATCTTGAAG
oCAH148 gcv FRT-Gm^R^-FRT R	cgttttttgaaaCGAATTAGCTTCAAAAGC
oCAH149 gcv 1kbDWN F	tgaagctaattcgTTTCAAAAAACGGCCTGCAAAAAGC
oCAH150 gcv 1kbDWN R	tcgctcgcgtatcggtgattcattcTCGCGCCTGGAAGTGAGC
oCAH151 gcv KO F	GTGCCAATACTACGCTCCG
oCAH152 gcv KO R	TCATGTATCGCTGATCGAGC
pCbbLS-inducible RubisCO construct and confirmation
oCAH149 pCAH01 R	TTCACTTTTCTCTATCACTGATAG
oCAH152 pCAH01 F	AAGCTTGACCTGTGAAGTG
oCAH644 *cbbL* F	gtgatagagaaaagtgaaATGGCTGTCAAAACATACAACGCGG
oCAH645 *cbbS* R	cttcacaggtcaagcttTCAGAAAAACGTCGCTACGGCG
oCAH172 pCAH01seq F	CCCGACACCATCGAATGGCCAGATG
oCAH173 pCAH01seq R	CAGGGCGCGTGGAGATCCGT

a Primer numbers in the Henard laboratory primer inventory at the University of North Texas. All others in this table represent primers from the Henard primer inventory at the National Renewable Energy Laboratory.

b Lowercase indicates homologous sequence for Gibson assembly.

### Isotopic elemental analysis.

Cultures for isotopic and elemental analysis were grown in sealed serum vials as described above with 20% CH_4_ in air (vol/vol). ^13^CO_2_ (Sigma) was added to the headspace via syringe to reach the indicated final concentrations, and cultures were incubated at 37°C with 200 rpm orbital shaking for 48 to 72 h. At indicated time points postinoculation, culture density was measured spectrophotometrically using a NanoDrop spectrophotometer (Thermo). Bacterial cells were then pelleted via centrifugation and freeze-dried prior to analysis. Isotopic and elemental analyses were conducted by combustion in a Flash 2000 elemental analyzer (Thermo) coupled with an isotope ratio mass spectrometer (Delta V; Thermo Scientific). Standards were used to account for linearity, drift, and isotopic discrimination.

### RNA sequencing.

M. capsulatus Bath transcription was evaluated during logarithmic growth in a continuous gas reactor as described above with 20% CH_4_/2% CO_2_ in air. Culture samples (2 ml; OD_600_ = 1) were pelleted by centrifugation, resuspended in 500 μl RNALater (Ambion), and stored at −20°C. Frozen samples were shipped to Genewiz (South Plainfield, NJ) for RNA isolation, library preparation, and sequencing. RNA was submitted to quality control analysis before rRNA depletion. Sequencing was performed using the Illumina HiSeq platform. Paired-end 150-bp Illumina reads were analyzed by Genewiz using their standard transcriptome sequencing (RNAseq) analysis pipeline. Reads were mapped to the *M. capsulatus* Bath reference genome (GenBank accession no. NC_002977.6), and transcript abundance was supplied as relative transcripts per million (TPM).

### ^13^C tracer analyses.

Sample preparation and gas chromatography-mass spectrometry (GC-MS) for amino acid isotopomer analysis were performed as previously reported (52). Liquid chromatography-tandem mass spectrometry (LC-MS/MS) for determination of 3-phosphoglycerate and ribulose-1,5-bisphosphate isotopomers was conducted as previously reported (53). For all isotopic tracer analyses, 30-ml cultures in 150 ml serum vials were inoculated at an OD_600_ of 0.1 and incubated with 20% CH_4_/8% ^13^CO_2_ in air (vol/vol). Samples for proteinogenic amino acid derivatization were prepared from 5 ml of logarithmically growing cultures at 24 h postinoculation. For determination of phosphorylated compounds, ^13^CO_2_ (8% final) was added via a syringe to cultures growing exponentially on 20% CH_4_ in air (OD_600_ = 0.4) and incubated at 37°C with 200 rpm orbital shaking for 4 h. After incubation, cultures were rapidly harvested by vacuum filtration using a Büchner funnel with a mixed cellulose ester (MCE) membrane filter (0.2 μM, 47 mm). Cells were washed with 5 ml ice-cold NMS medium before the filter was immediately transferred using forceps to a 15-ml centrifuge tube and flash frozen in liquid nitrogen. Frozen cells/membrane were stored at −80°C until metabolite extraction.

### Data availability.

Plasmids, bacterial strains, and other data will be made available upon reasonable request.
