# Supplementary material for: Ribulose-1,5-Bisphosphate Carboxylase/Oxygenase (RubisCO) Is Essential for Growth of the Methanotroph Methylococcus capsulatus Strain Bath
Source: Appl Environ Microbiol. 2021 Aug 26;87(18):e00881-21. doi: 10.1128/AEM.00881-21 (PMC8388818; doi:10.1128/AEM.00881-21)
Supplement: Supplemental file 1 — Fig. S1 and Fig. S2, legends to Tables S1 and S2. Download AEM.00881-21-s0001.pdf, PDF file, 0.3 MB [file aem.00881-21-s0001.pdf]

Supplemental Material Henard et al., Ribulose-1,5-bisphosphate carboxylase/oxygenase (RubisCO) is essential for growth of the methanotroph *Methylococcus capsulatus.*

Supplemental Figures

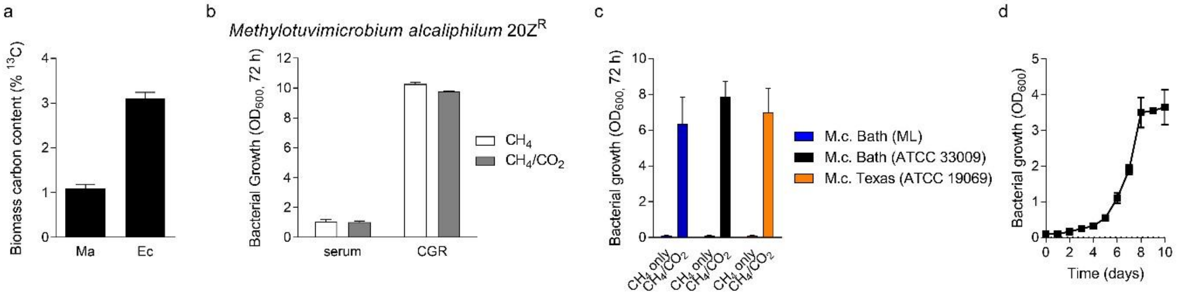

Figure S1. a) The percent of *Methylotuvimicrobium alcaliphilum* 20Z^R (Ma) and *Escherichia coli* (Ec) biomass derived from $^{13}CO_2$ determined by isotopic elemental analysis after 72 h cultivation with 20% unlabeled CH_4 and 8% $^{13}CO_2$ (Ma) or 8% $^{13}CO_2$ only (Ec) in the gas phase of serum vials. b) Growth of *M. alcaliphilum* 20Z^R in a sealed serum vial or a continuous gas reactor (CGR) with 20% CH_4 in air only (white bar) or supplemented with 2% CO_2 (gray bar). c) Culture density (OD_{600}) of two independent *Methylococcus capsulatus* Bath strains obtained from Mary Lidstrom's laboratory (ML) or ATCC (strain 33009) and *M. capsulatus* Texas from ATCC (strain 19069) after 72 h of growth in a CGR supplied with 20% CH_4 in air (CH_4 only) or 20% CH_4 and 2% CO_2 in air (CH_4/CO_2) at 1 volume gas mixture/volume medium/minute.
d) *M. capsulatus* growth in a CGR supplied with 20% CH_4 in air at 0.1 volume gas mixture/volume medium/minute. The data represent the mean ± standard deviation of 4-6 biological replicates from two independent experiments.

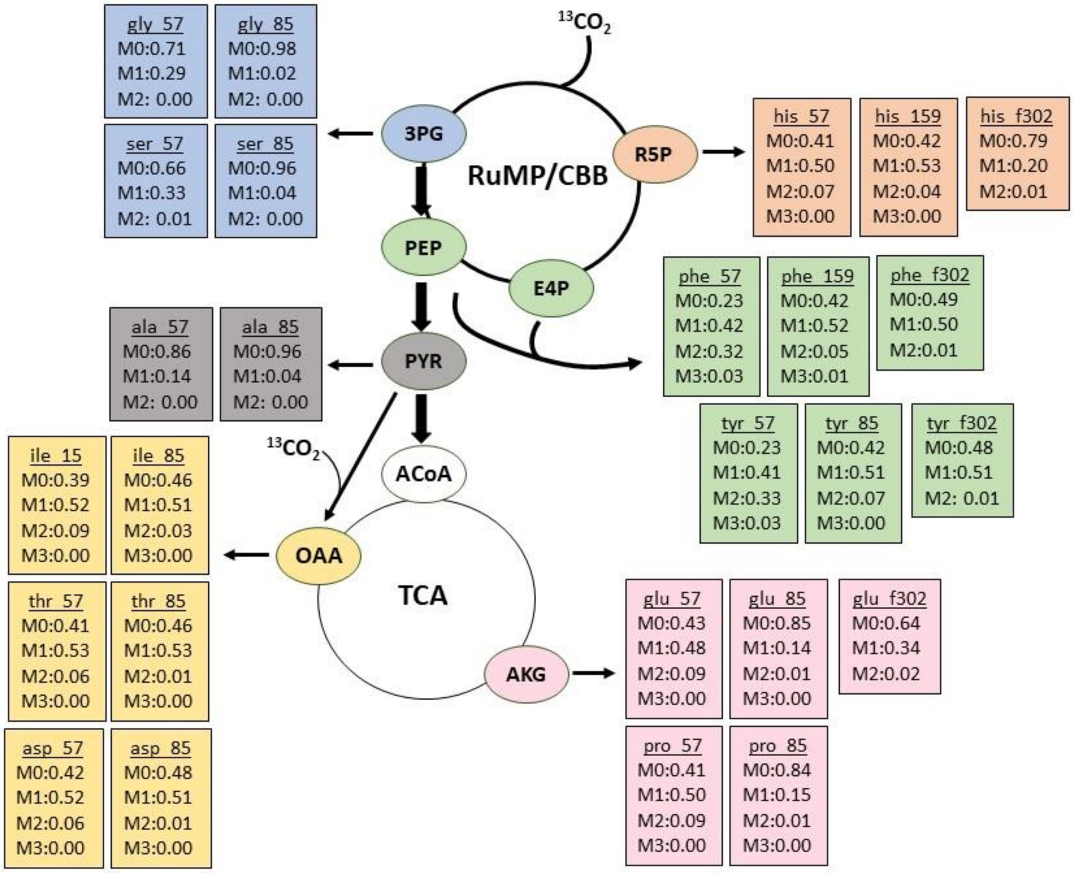

Figure S2. Amino acid mass isotopomer distributions after $^{13}CO_2$ isotopic labeling. Amino acids and their respective metabolite precursors are color-coded: glycine and serine from 3-phosphoglycerate (3PG, light blue); histidine from ribose-5-phosphate (R5P, orange); phenylalanaine and tyrosine from phosphoenolpyruvate (PEP, green) and erythrose-4-phosphate (E4P, green); alanine from pyruvate (PYR, gray); isoleucine, threonine, and aspartate from oxaloacetate (OAA, yellow); and glutamate and proline from alpha ketoglutarate (AKG, pink). The data represent the mean mass isotopomer distribution vector of three biological replicates.

Supplemental Tables

Table S1. ***M. capsulatus*** **relative transcripts per million (TPM) during cultivation with continuous 20%CH_4/2% CO_2 in air.**
See accompanying Excel spreadsheet.

Table S2. Isotopomer analysis.
See accompanying Excel spreadsheet.
